# Effect of dapagliflozin, a sodium-glucose co-transporter 2 inhibitor, on ventricular repolarization electrocardiographic parameters in type 2 diabetes patients: DAPA - ECG study

**DOI:** 10.3389/fcdhc.2025.1537005

**Published:** 2025-03-31

**Authors:** Rodrigo Noronha Campos, Dalmo Antônio Ribeiro Moreira, Gabriel Mostaro Fonseca

**Affiliations:** ^1^ Dante Pazzanese Institute of Cardiology (IDPC), São Paulo, Brazil; ^2^ Beneficência Portuguesa Hospital, São Paulo, Brazil

**Keywords:** type 2 diabetes, dapagliflozin, ventricular repolarization, electrocardiogram, cardiac arrhythmias

## Abstract

**Background:**

Type 2 diabetes (T2DM) is a chronic metabolic disorder that affects approximately 10.5% of the world’s population and is an independent risk factor for cardiovascular complications, including sudden cardiac death (SCD). Inhibitors of sodium-glucose co-transporter type 2 (iSGLT2), particularly dapagliflozin, have emerged as promising treatments in patients with T2DM and with heart failure and chronic kidney disease, demonstrating the ability to significantly reduce major cardiovascular events. However, the exact mechanisms that promote the observed benefits are still not fully understood.

**Objective:**

In this study, we sought to understand the mechanisms associated with the benefits of dapagliflozin by evaluating various electrophysiological parameters of the electrocardiogram (ECG) in patients with T2DM. A randomized, multicenter, prospective study with 174 patients with T2DM divided into two groups: one receiving dapagliflozin plus optimized guideline directed medical therapy (GDMT) and the other optimized GDMT without SGLT2 inhibitors. Clinical, electrocardiographic, laboratory, and echocardiographic evaluations were performed initially and after three months. Descriptive and inferential statistics were used, with a significance level of 0.05.

**Result:**

This study shows that in patients treated with dapagliflozin plus GDMT, a significant reduction in the duration of the interval from the peak of the T wave to the end of the T wave (TpTe), the QTc interval, and the ratio between the TpTe/QT intervals was observed, with no change in other electrocardiographic variables such as QT interval dispersion, JT peak interval, or changes in the QRS complex and T wave axes (QRS-T angle).

**Conclusion:**

In patients with T2DM, dapagliflozin significantly shortened the TpTe and QTc intervals, as well as the TpTe/QT ratio. These results suggest a reduction in ventricular electrical remodeling, highlighting a potential cardioprotective effect of dapagliflozin.

**Clinical trial registration:**

https://clinicaltrials.gov/study/NCT06721442, identifier NCT06721442.

## Introduction

Type 2 diabetes mellitus (T2DM) is a chronic metabolic disorder that affects approximately 10.5% of the world’s population and is an independent risk factor for cardiovascular complications, including sudden cardiac death (SCD) (1). Individuals with T2DM have twice the risk of cardiovascular death (CVD) compared to non-diabetics, even under glycemic control and management of comorbidities such as hypertension and dyslipidemia (2). SCD is three to eight times more prevalent in diabetic patients, often occurring independently of existing coronary artery disease (CAD), which indicates that other factors contribute to the high cardiovascular risk in this population (3–5).

Inhibitors of sodium-glucose co-transporter type 2 (iSGLT2), particularly dapagliflozin, have emerged as promising treatments in patients with T2DM and with heart failure and chronic kidney disease, demonstrating the ability to significantly reduce major cardiovascular events (6). A previous study (the DAPA-HF study) has demonstrated a reduction in the composite endpoint of worsening heart failure (HF) or CVD in patients with HF and reduced ejection fraction (HFrEF) (7). The sub-analysis of this study revealed that dapagliflozin was associated with a 21% reduction in the combined endpoint of first occurrence of serious ventricular arrhythmias, resuscitated cardiac arrest or SCD, suggesting that the impact of this drug may extend beyond hemodynamic changes (8). However, the exact mechanisms that promote the observed benefits are still not fully understood.

Electrocardiogram data from patients with T2DM often show abnormalities in ventricular repolarization, such as prolongation of the QT and QTc intervals, as well as increased dispersion of the QT interval (9, 10). Currently, emerging indices, such as the TpTe interval and the TpTe/QT ratio, are proposed as more accurate metrics for assessing the dispersion of ventricular repolarization and vulnerability to ventricular arrhythmias compared to traditional metrics such as the QT interval. Studies suggest that prolongation of the TpTe interval is independently associated with a higher risk of SCD, even in patients with normal QTc (11–13).

The TpTe interval reflects the phase 3 of ventricular repolarization, which is dominated by the activation of potassium outflow currents, including the fast current (I_Kf_) and the slow current (I_Ks_) (11, 14). Alterations in the expression or function of these currents are present in patients with T2DM and can prolong the TpTe interval, increasing the dispersion of ventricular repolarization and susceptibility to serious arrhythmias (11, 12, 15–17).

In this study, we sought to understand the mechanisms associated with the benefits of dapagliflozin by evaluating various electrophysiological parameters of the ECG in patients with T2DM, such as the duration of the TpTe, QT and QTc intervals, the ratio between the TpTe/QT intervals, the dispersion of the QT interval duration, the JT peak (JTp) interval, and the differences between the electrical axes of the QRS complexes and T waves (QRS-T angle) and heart rate.

## Methods

### Study design

From January to December 2023, a total of 174 T2DM patients were randomized to receive dapagliflozin+GDMT or GDMT only at three different centers in the city of São Paulo, Brazil, namely Dante Pazzanese Cardiology Institute (IDPC), Beneficência Portuguesa Hospital of São Paulo (BP/São Paulo), and National Diabetes Care Association (ANAD) (see **Table 1**).

**Table 1:** Demographic and clinical characteristics of the patients at the start of the study.

	Dapagliflozin+GDMT	GDMT	p-value
Number	87	87	
Age in years (mean ± SD)	71.02 ± 11.1	71.62 ± 11.26	0,821
Women, number (%)	47 (54.0)	30 (34.5)	0,009
Race, number (%)			0,426
White	68 (78.2)	75 (86.2)	
Black	7 (8.0)	6 (6.9)	
Asian	8 (9.2)	5 (5.7)	
Brown	4 (4.6)	1 (1.1)	
Disease history, no. (%)			
Smoking	8 (9.2)	6 (6.9)	0,577
Hypertension	66 (75.9)	64 (73.6)	0,727
Dyslipidemia	66 (75.9)	63 (72.4)	0,603
Heart Failure*	2 (2.3)	3 (3.4)	0,651
Clinical data (mean ± SD)			
BMI (kg/m²)	29.79 ± 4.29	28.24 ± 4.89	0,065
SBP (mmHg)	129 ± 18	126 ± 21	0,197
Heart Rate (bpm)	72 ± 14	75 ± 11	0,641
Laboratory tests (mean ± SD)			
HbA1c (%)	6.46 ± 0.88	6.58 ± 0.93	0,324
Potassium	4.48 ± 0.41	4.47 ± 0.45	0,694
LDL cholesterol	86.46 ± 36.33	92.96 ± 38.03	0,228
CRP	0.54 ± 1.32	0.36 ± 0.95	0,055
GFR	68.30 ± 17.33	68.43 ± 15.24	0,935
Antiarrhythmic medications, no. (%)			
Propafenone	1 (1.1)	0 (0.0)	–
Amiodarone	1 (1.1)	2 (2.3)	–
Sotalol	0 (0.0)	0 (0.0)	–
Diltiazem or Verapamil	0 (0.0)	1 (1.1)	–
Beta-blocker	25 (28.7)	32 (36.8)	0,258
General medications, number (%)			
ACEI	11 (12.6)	4 (4.6)	0,059
ARB	23 (26.4)	25 (28.7)	0,734
Sacubitril-valsartan	2 (2.3)	2 (2.3)	–
MRA	0 (0.0)	2 (2.3)	–
Antidiabetic medications, no. (%)			
Sulfonylureas	18 (20.7)	9 (10.3)	0,093
Metformin	80 (92.0)	77 (88.5)	0,444
Thiazolidinediones	4 (4.6)	2 (2.3)	0,682
iDPP4	2 (2.3)	1 (1.1)	–
Electrocardiographic data (mean ± SD)			
Heart Rate (bpm)	74 ± 13	73 ± 10	0,995
P wave (D2)	114.63 ± 11.52	111.38 ± 12.85	0,075
PR interval (D2)	174.47 ± 27.89	166.26 ± 20.38	0,430
QRS interval (V5)	89.28 ± 9.32	87.70 ± 11.98	0,499
QTc interval (V5)	424.07 ± 30.76	424.89 ± 25.38	0,525
Tpeak/Tend	76.87 ± 12.47	73.67 ± 11.42	0,444
Echocardiographic data (mean ± SD)			
End-diastolic diameter (mm)	49.0 ± 5.0	47.0 ± 5.0	0,067
Ventricular mass index	81.75 ± 24.92	78.95 ± 20.75	0,061
LVEF (%)	64.63 ± 7.77	66.27 ± 6.54	0,232
Atrial diameter (mm)	37.0 ± 5.0	36.0 ± 5.0	0,820

GDMT, guideline directed medical therapy for type 2 diabetes mellitus without dapagliflozin; BMI, body mass index; SBP,: systolic blood pressure; DBP, diastolic blood pressure; HbA1c, glycated hemoglobin; CRP, C-reactive protein; GFR, glomerular filtration rate; *Clinical signs of heart failure by Framingham score; ACEI, angiotensin-converting enzyme inhibitor; ARB, angiotensin II receptor blocker; MRA, mineralocorticoid receptor antagonist; iDPP4, dipeptidyl peptidase 4 inhibitors; LVEF, left ventricular ejection fraction.

This was a randomized, multicenter, prospective study. All participants were encouraged to actively manage cardiovascular risk factors such as hypertension, dyslipidemia, obesity, and smoking, in line with current clinical guidelines to optimize health care (18, 19). The study was approved by the National Committee for Ethics in Research (CONEP) in Brazil under number CAAE: 39884520.7.1001.5462.

### Sample

The sample size calculation was based on the change in the TpTe interval, which was established as the primary endpoint of the study. This decision was supported by findings from a pilot study suggesting that dapagliflozin may confer beneficial effects on this parameter. Based on estimates from the pilot study for the intervention group and a population study for the control group, we calculated that, to detect an intergroup difference of 8.17 ms with a significance level of 5% and a power of 90%, 87 participants would be required per group, resulting in a total of 174 patients (20, 21).

### Inclusion and exclusion criteria

This study included adult patients (≥ 18 years) with T2DM, a BMI of 45 or less, a glomerular filtration rate (GFR) of at least 30 ml/min/1.73 m². Patients were excluded if they had a QRS interval > 120 ms, were taking insulin or weight-loss medication in the three months prior to the study, were pregnant or breastfeeding, had atrial fibrillation, a pacemaker or cardiac resynchronization therapy (CRT), had uncontrolled blood glucose (glucose > 240 mg/dL after fasting for 8 h), had significant liver disease, or had cardiac surgery or angioplasty planned within three months of starting the study. All patients signed an informed consent form.

### Study procedures

**Figure 1:**
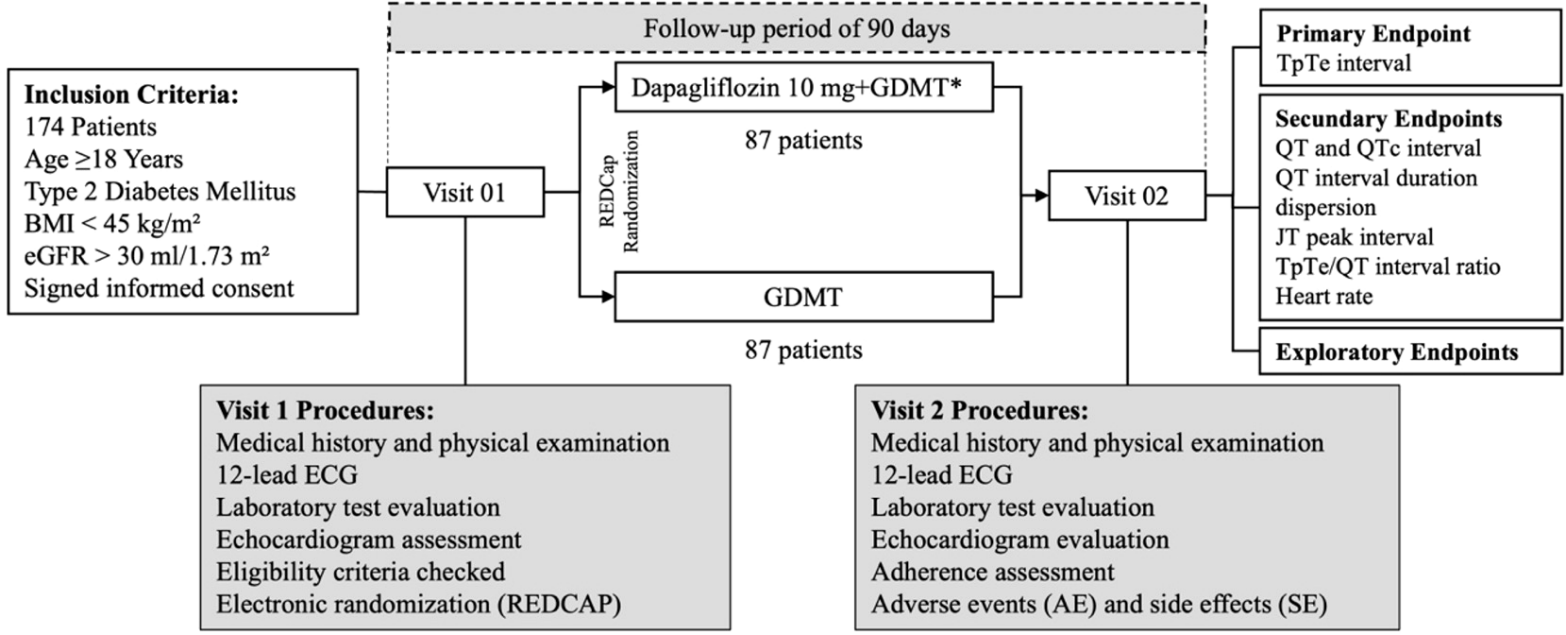
Screening flowchart of the study population. BMI, body mass index; eGFR, estimated glomerular filtration rate; GDMT, guideline-directed medical therapy.

Participants were randomized in a 1:1 ratio to receive either 10 mg of dapagliflozin combined with GDMT or GDMT alone for T2DM, excluding SGLT2 inhibitors. Randomization was performed using the RedCap software (22) to ensure balanced group allocation. Initial evaluations were conducted in an outpatient clinic and included clinical examination, electrocardiogram, laboratory tests, and echocardiography. After three months, participants returned for a follow-up visit, which included a medical assessment and an electrocardiographic evaluation. **Figure 1** presents the study population screening flowchart.

### Measurement of electrocardiographic intervals

The ECGs were recorded using an ECG monitor (Micromed, Brasília, DF, Brazil), with settings of 25 mm/s and 10 mm/mV. Interval measurements were taken manually using Wincardio software (Micromed). An interobserver evaluation with two independent evaluators, performing the kappa test on 30% of the participants, revealed a kappa coefficient of 0.8, confirming the consistency and reliability of the electrocardiographic measurements.

The TpTe interval, measured in lead V5, represents the time between the peak and the end of the T wave on the electrocardiogram, used to assess the transmural dispersion of left ventricular repolarization. The QRS complex, also measured in lead V5, from the start of the Q wave to the end of the S wave, was used to calculate other parameters, including the difference between the QRS complex and T wave angles (QRS-T angle). The QT interval, measured from the start of the QRS complex to the end of the T wave, was corrected using Bazett’s formula (QTc = QT/√RR interval) (23). The J-Tp interval (JTp) was determined from the J point to the apex of the T wave. QT interval dispersion (QTd) was assessed as the difference between the longest and shortest QT duration in the 12 ECG leads. The QRS-T planar angle was calculated by the difference between the electrical axes of the QRS and T complexes in the frontal plane leads using My EKG through a specific computational algorithm (24).

### Statistical analysis

The data were described as mean and standard deviation for continuous variables and frequencies for categorical variables. Homogeneity between the groups was assessed using appropriate statistical tests such as Chi-square, Fisher’s, Student’s *t*, or Mann-Whitney tests. A linear mixed-effects model was used to compare the outcomes, considering treatment, time, and the interaction between them as fixed effects and the patient as a random effect. In order to assess the homogeneity of the results, the difference between the outcomes at baseline and endpoint and the importance of the interaction between the patients’ characteristics were calculated. Treatment was assessed using a linear fixed-effects model. The significance level adopted was 0.05 and 95% confidence intervals (95% CI) and graphical representations were used when necessary. The analyses were conducted using IBM SPSS Statistics for Windows version 25.0 (IBM Corp, Armonk, NY, USA) and R 4.3.2 software (R Foundation for Statistical Computing, Vienna, Austria), with data management conducted using REDCap software (22). To identify factors that could modulate the response to treatment with dapagliflozin, sensitivity analysis was performed, which involved stratifying patients based on predefined clinical and demographic characteristics. The clinical impact of the results of dapagliflozin on ventricular repolarization was determined by Cohen’s d.

## Results

### Demographic and clinical characteristics of the study patients

A total of 174 patients were equally distributed between two groups: Dapagliflozin+GDMT and GDMT. Patient characteristics and drug therapies were similar between the groups at the start of the study (**Table 1**), except for gender distribution, with a significantly higher proportion of women in the treatment group (p = 0.009). All outcome analyses were adjusted for gender, and the p-value of the interaction is presented in the results. The average period of treatment with dapagliflozin was 95 days, with an adherence rate of 98.56%.

### Primary endpoint

**Figure 2:**
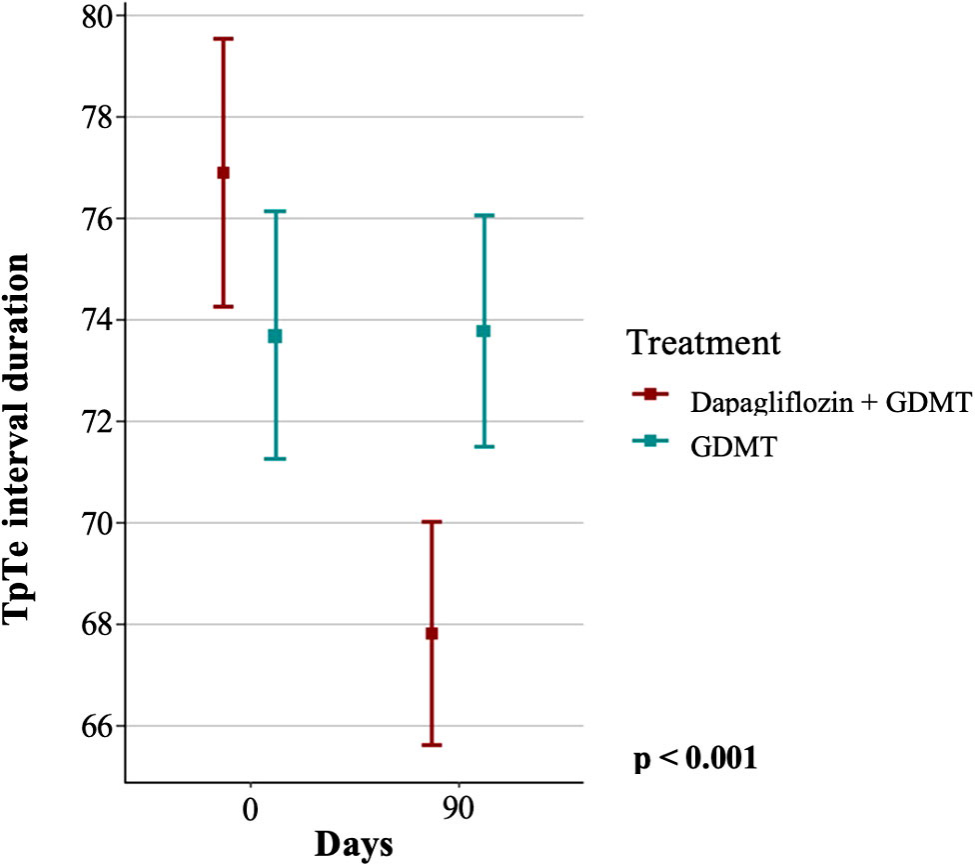
Duration of the TpTe interval in the dapagliflozin+GDMT and GDMT groups at baseline and after three months. GDMT, guideline-directed medical therapy.

The primary endpoint, based on the change in the duration of the TpTe interval in diabetic patients, was observed in the dapagliflozin group but not in the GDMT group, as shown in **Figure 2**.

Initially, the GDMT group had a mean TpTe interval of 73.67 ± 11.42 ms, which remained practically unchanged at follow-up, with a mean of 73.76 ± 10.72 ms. In contrast, the group treated with dapagliflozin+GDMT showed a significant reduction in the mean TpTe interval from 76.87 ± 12.47 ms at baseline to 67.8 ± 10.34 ms after treatment (p < 0.001).

### Secondary endpoints

**Figure 3:**
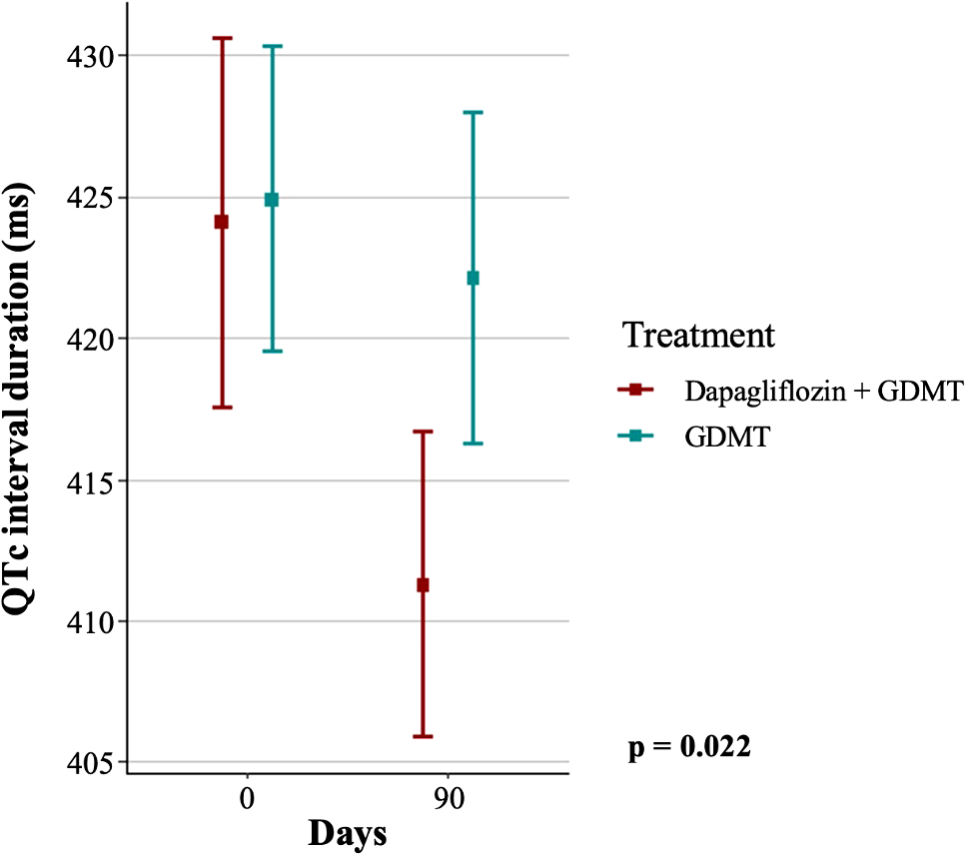
Duration of the QTc interval in the dapagliflozin+GDMT and GDMT groups at the start of treatment and after three months. GDMT, guideline-directed medical therapy.

In the GDMT group, the mean duration of the QT interval was 387.97 ± 31.65 ms at baseline and 390.21 ± 30.13 ms at the three-month follow-up, whereas in the dapagliflozin+GDMT group the mean values were 385.63 ± 34.69 ms at baseline and 382.09 ± 31.5 ms after treatment (p = 0.117). In addition, the baseline mean for the QTc interval in the GDMT group was 424.89 ± 25.38 ms, with only a slight reduction to 422.11 ± 27.58 ms at follow-up, whereas mean QTc interval decreased significantly from 424.07 ± 30.76 ms at baseline to 411.26 ± 25.31 ms at three months in the dapagliflozin+GDMT group (p = 0.022, **Figure 3**).

**Figure 4:**
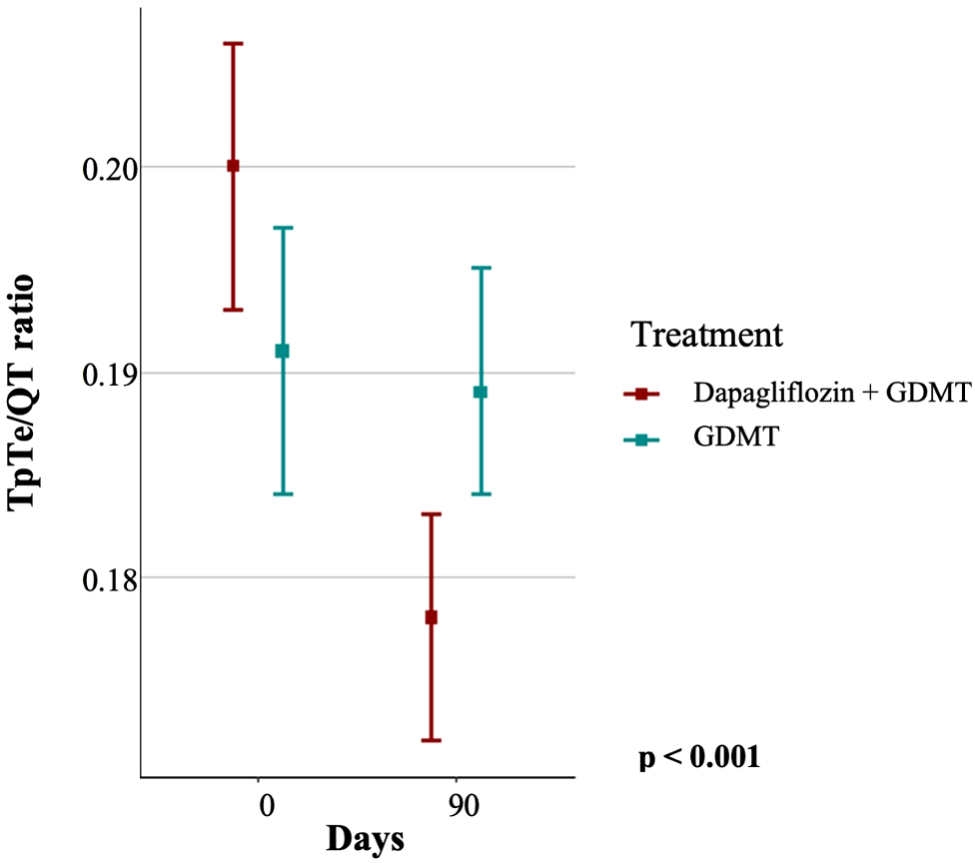
Relationship between the TpTe and QT intervals in the dapagliflozin+GDMT and GDMT groups at baseline and after three months. GDMT, guideline-directed medical therapy.

Analysis of the TpTe/QT ratio, i.e., the relationship between the TpTe endpoint and the QT interval, revealed that the mean ratio in the GDMT group was 0.19 ± 0.03 at baseline and remained unaltered at follow-up. Conversely, the mean TpTe/QT ratio in the dapagliflozin+GDMT group decreased significantly from 0.2 ± 0.03 at baseline to 0.18 ± 0.02 at the end of the study compared to the GDMT group (p < 0.001, **Figure 4**).

Analysis of the dispersion of the QT interval duration, the peak J-T interval (JTp), the differences between the electrical axes of the QRS complexes and T waves (QRS-T angle), and the heart rate between the groups at baseline and at follow-up of the study reveals no significant differences between the two groups (**Table 2**).

**Table 2:** Electrocardiographic parameters and heart rate in the dapagliflozin+GDMT and GDMT groups at baseline and after three months.

		Baseline		Three months		p-value
		Mean	SD	Mean	SD	
QT interval dispersion	GDMT	15.89	9.69	14.94	10.21	0.568
	Dapagliflozin+ GDMT	17.89	11.37	15.78	13.14	
JTp	GDMT	226	32	228	30	0.576
	Dapagliflozin+ GDMT	222	39	220	42	
QRS-T angle	GDMT	33.13	29.87	29.87	28.23	0.588
	Dapagliflozin+ GDMT	53.89	58.27	47.85	44.53	
HR (bpm)	GDMT	73	10	71	11	0.595
	Dapagliflozin+ GDMT	74	13	71	13	

GDMT, guideline directed medical therapy for type 2 diabetes mellitus without dapagliflozin; SD, standard deviation; JTp, interval between the J point and the peak of the T wave; QRS-T angle, difference between the QRS complex angle and that of the T wave; HR, heart rate on the electrocardiogram.

### Exploratory outcomes

There were no significant mean differences between the dapagliflozin+GDMT and GDMT groups at baseline and follow-up in body mass index (BMI) (GDMT: from 28.24 ± 4.89 to 27.76 ± 4.94 kg/m²; dapagliflozin+GDMT: from 29.79 ± 4.29 to 29.06 ± 4.24 kg/m²; p = 0.441), systolic blood pressure (SBP) (GDMT: from 126 ± 21 to 125 ± 18 mmHg; dapagliflozin+GDMT: from 129 ± 18 to 130 ± 19 mmHg; p = 0.743), and diastolic blood pressure (DBP) (GDMT: from 72 ± 13 to 73 ± 17 mmHg; dapagliflozin + GDMT: from 78 ± 12 to 76 ± 11 mmHg; p = 0.323).

At baseline, the mean blood glucose levels were 123.97 ± 29.22 mg/dL in the GDMT group and 117.43 ± 27.11 mg/dL in the dapagliflozin+GDMT group. At the end of the study, these averages were reduced to 109.15 ± 29.96 mg/dL in the GDMT group and 113.9 ± 19.78 mg/dL in the dapagliflozin+GDMT group. Despite the reduction observed in both groups, the decrease in blood glucose levels was statistically significant only in the GDMT group (p = 0.008). In addition, mean HbA1c was 6.58 ± 0.93% in the GDMT group and 6.46 ± 0.88% in the dapagliflozin+GDMT group at baseline and declined to 6.13 ± 0.47% and 6.39 ± 1.16% in the GDMT and dapagliflozin+GDMT groups at follow-up, respectively, with a significant reduction in HbA1c in the GDMT group (p = 0.008).

### Primary outcome, according to predefined subgroups

**Figure 5:**
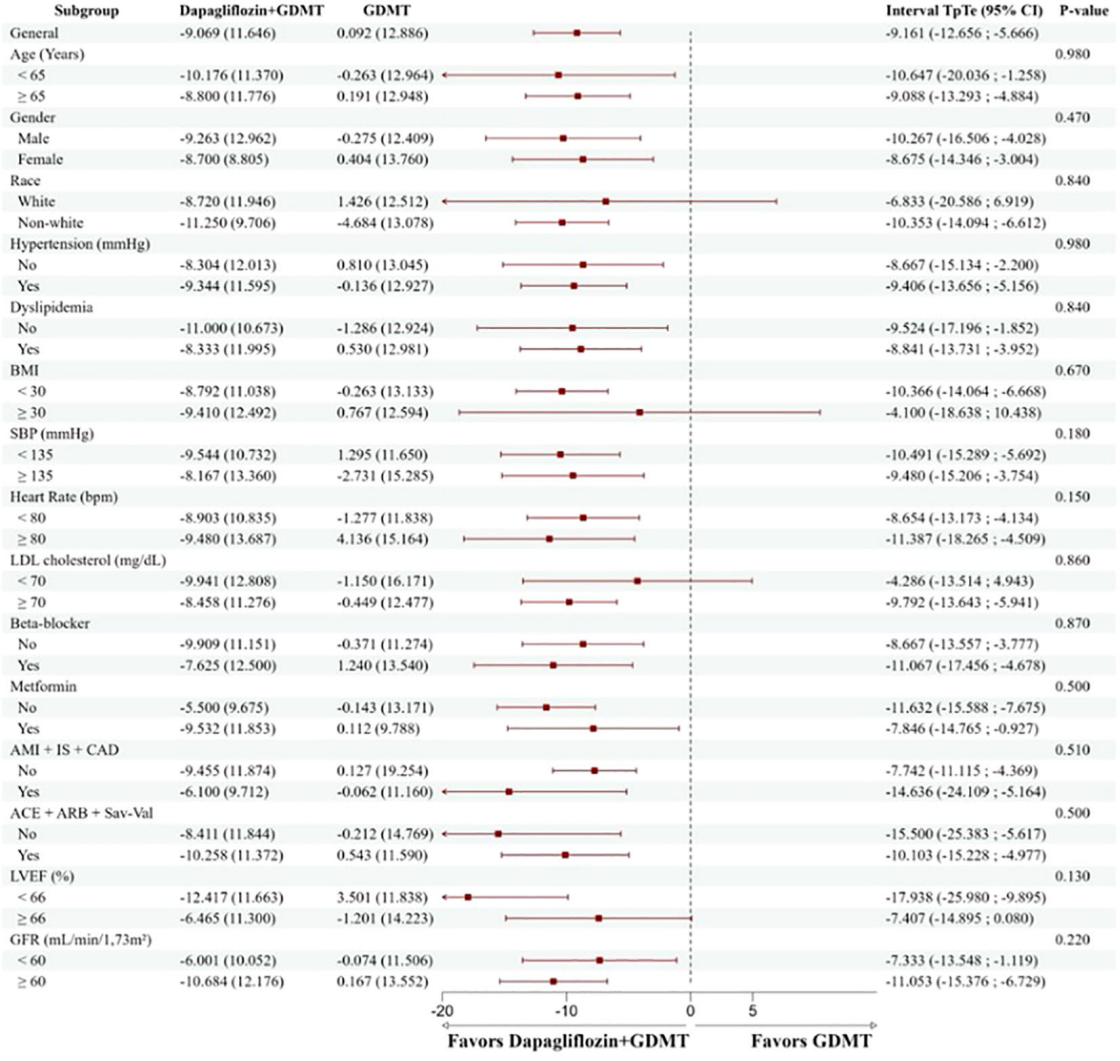
Reduction of the TpTe interval according to the predefined subgroups. The group treated with dapagliflozin+GDMT had a consistent and positive result in the primary endpoint in all predefined subgroups, regardless of characteristics such as gender, race, age, health conditions, medication use, and other relevant clinical factors. BMI, body mass index; SBP, systolic blood pressure; AMI, acute myocardial infarction; CVA, ischemic stroke; CAD, significant coronary atheromatosis (greater than 50%); ACEI, angiotensin-converting enzyme inhibitor; ARB, angiotensin II receptor blocker; Sac-Val, Sacubitril-Valsartan; LVEF, left ventricular ejection fraction; GFR, glomerular filtration rate.

The outcome of the primary endpoint in the group treated with dapagliflozin+ GDMT was consistent among the predefined subgroups, including gender, race, age, hypertension, dyslipidemia, BMI, blood pressure values, heart rate, LDL cholesterol, use of beta-blockers, metformin, presence of established atherosclerotic disease (AMI, stroke, and CAD), use of renin-angiotensin-aldosterone system blockers (ACEI, ARB, or sacubitril-valsartan), LVEF, and GFR (**Figure 5**).

### Effect size calculation

Quantification of the effect size for the primary endpoint of reducing the TpTe interval yielded a Cohen’s d value of 0.746, indicating an effect of considerable magnitude and suggesting that the intervention studied has a significant clinical impact in reducing the TpTe interval.

## Discussion

This study shows that in the group of patients treated with dapagliflozin+GDMT there was a significant reduction in the duration of the TpTe interval, the QTc interval, and the ratio between the TpTe/QT intervals, with no change in other electrocardiographic variables such as QT interval dispersion, JT peak interval, or changes in the QRS complex and T wave axes (QRS-T angle). Our findings indicate that the benefits observed were caused by dapagliflozin and are in line with recent studies which have shown a reduction in mortality in patients treated with iSGLT2 (8).

**Figure 6:**
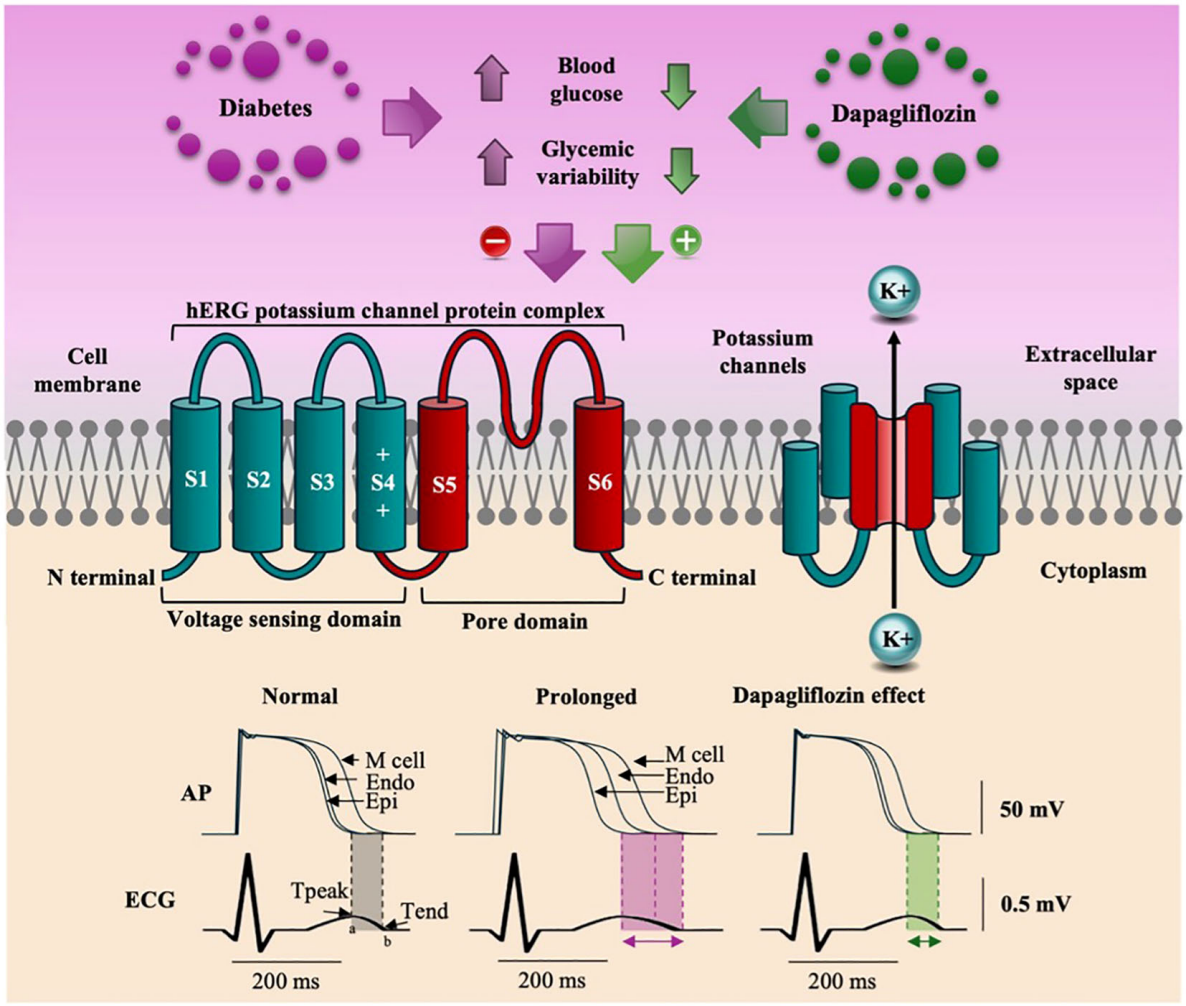
The figure illustrates that hyperglycemia and glycemic variability reduce the expression of the HERG gene, decreasing potassium efflux and prolonging ventricular repolarization (TpTe). Dapagliflozin increases HERG gene expression, normalizes potassium efflux, and significantly reduces the TpTe interval.

Ventricular repolarization involves voltage gradients between the epicardium, the M cells, and the endocardium. The end of epicardial repolarization coincides with the peak of the T wave (Tpeak), whereas the end of M-cell repolarization coincides with the end of the T wave (Tend), and endocardial repolarization occurs in the intermediate portion between the peak and the end of the T wave (25). Electrical remodeling, evidenced by prolongation of the QT interval, occurs in approximately 25% of patients with T2DM and is influenced by acute and chronic hyperglycemia, high HbA1c levels, and glycemic variability (26–29). These factors negatively affect the activity of the hERG gene (Human Ether-à-go-go-Related Gene), responsible for the formation of potassium channels (**Figure 6**), resulting in a delay in the conduction of potassium currents (I_K_) and, consequently, prolonging the QT interval, especially in phase 3 of the action potential (30).

The TpTe interval represents the transmural dispersion of ventricular repolarization, indicating the moment when the epicardium is already repolarized while the M cells are still completing their repolarization. Under abnormal conditions, these exacerbated differences in the duration of action potentials can favor the onset of serious arrhythmias such as ventricular tachycardia and ventricular fibrillation (12, 25). Our results indicate that, in patients with T2DM, dapagliflozin attenuates electrical remodeling, as evidenced by a reduction in the TpTe interval. A study in an animal model showed that dapagliflozin acts by increasing voltage-dependent potassium currents (I_K_) and restoring mitochondrial activity. By normalizing the efflux of ions by the fast and slow rectifying potassium currents (I_Kf_ and I_Ks_, respectively), dapagliflozin accelerates the repolarization of myocardial cells, attenuating the abnormal prolongation of repolarization (31, 32).

Cardiac dysfunction in T2DM, even in the absence of other conditions, suggests the presence of an intrinsic diabetic cardiomyopathy. This condition is characterized by electrical and metabolic changes that result in prolongation of the QT interval (30). Safety studies of the drug indicate that dapagliflozin does not significantly alter the QTc interval in healthy individuals, even at supratherapeutic doses, reinforcing that its benefits observed in this study would be linked to the correction of the abnormal prolongation of ventricular repolarization commonly present in patients with diabetes (33).

The TpTe/QT ratio is used as an index of myocardial vulnerability to ventricular arrhythmias, indicating the ratio between the dispersion of repolarization and the total time of ventricular electrical activity. The TpTe interval can vary with heart rate; conversely, the TpTe/QT ratio tends to be a more constant variable (11, 34). Previous studies in individuals with T2DM have failed to show that glycemic control using other oral antidiabetics (without SGLT2 inhibitors) improves the heterogeneity of ventricular repolarization (35). However, the present study observed a significant reduction in the TpTe/QT ratio with dapagliflozin, suggesting greater homogenization of ventricular repolarization caused by the shortening of the TpTe interval.

The JTp interval represents the initial phase of ventricular repolarization on the ECG and involves L-type sodium and calcium currents, unlike the TpTe interval, whose substrate is potassium currents (36). There was no significant difference in the behavior of the JTp interval in this study between the groups, suggesting a lesser influence of sodium and calcium channels on the attenuation of the QTc interval. Dapagliflozin has been shown to normalize potassium currents in animal model studies without significantly altering calcium channels, suggesting that its beneficial effects are mainly due to increased ion efflux through potassium channels, which is essential for maintaining myocardial electrical homeostasis (31).

Our findings strongly indicate that dapagliflozin attenuated the prolongation of ventricular repolarization, regardless of body weight changes, potassium levels, estimated glomerular filtration rate, C-reactive protein, echocardiographic parameters and was consistent in the different subgroups predefined in the study, suggesting that the drug may benefit a wide variety of patients with T2DM. These benefits have been widely documented in pivotal and extended follow-up studies (27, 37–41). In the current study, both groups showed an improvement in glycemic control and HbA1c, but there was a more evident reduction in the control group. Despite this, the group treated with dapagliflozin+GDMT showed a significant reduction in the TpTe and QTc intervals and in the TpTe/QT ratio, indicating that the benefits of dapagliflozin in correcting electrical remodeling in patients with T2DM are linked to mechanisms not associated with correcting glycemic levels (31, 32, 42).

## Study limitations

The application of specific inclusion and exclusion criteria restricted the generalizability of the findings because the data were collected exclusively from individuals with T2DM. Nevertheless, these criteria ensured the homogeneity of the sample and the relevance of the results for this specific population. Although the open design of the study and the lack of blinding may have influenced the behavior of the participants, strict clinical control, the use of standardized assessment criteria, and rigorous statistical analysis helped to minimize potential biases.

## Conclusion

In this study, we observed that patients treated with dapagliflozin showed a significant improvement in ventricular repolarization, demonstrated by a decrease in the TpTe and QTc intervals and a reduction in the TpTe/QT ratio. These results suggest that dapagliflozin can minimize the risk of severe ventricular arrhythmias and SCD by attenuating prolonged ventricular repolarization and promoting more homogeneous repolarization. The benefits observed occurred regardless of glycemic control, were noticeable over three months of treatment, and were consistent for all the subgroups analyzed. **Figure 6** presents our proposal for the mechanism by which dapagliflozin corrects the electrical remodeling characteristic of patients with T2DM, allowing for greater potassium efflux by IK currents.

This study contributes to the understanding of the cardiovascular benefits of dapagliflozin in patients with T2DM, reinforcing its role not only in glycemic management, but also as a cardioprotective agent, offering a promising perspective for reducing cardiovascular morbidity and mortality in this population.

## Data Availability

The original contributions presented in the study are included in the article/supplementary material. Further inquiries can be directed to the corresponding author.
